# Lethal and Sub-Lethal Effects of Organic-Production-Approved Insecticides and Fungicides on the Predator *Macrolophus pygmaeus* (Rambur) (Hemiptera: Miridae)

**DOI:** 10.3390/insects14110866

**Published:** 2023-11-09

**Authors:** Petri C. Betsi, Dionysios Ch Perdikis

**Affiliations:** Laboratory of Agricultural Zoology and Entomology, Agricultural University of Athens, Iera Odos 75, 118 55 Athens, Greece

**Keywords:** IPM, pesticide, partial prey consumption, sub-lethal effects, side-effects

## Abstract

**Simple Summary:**

*Macrolophus pygmaeus* is an important natural enemy of major tomato insect pests; however, its beneficial role may be negatively impacted by insecticides and fungicides. Studies on lethal and sub-lethal effects of widely used insecticides and fungicides on this predator are limited. This predator may slightly, partially or fully consume its prey, and this study examined the effects of pesticides on each of these predatory behaviors as a means of identifying in more detail the influences of pesticides on the activity of this predator. The effects of paraffin oil, copper hydroxide, copper oxychloride, wettable sulfur, *Beauveria bassiana* and deltamethrin, as a toxic reference treatment, were examined. The results showed that copper hydroxide and *B. bassiana* may cause significant adverse effects on the survival of *M. pygmaeus* depending on their application. In addition, the sub-lethal effects on the predatory behavior of *M. pygmaeus*, as shown by variation in the percentages of prey slightly, partially and fully consumed, were differentiated among the pesticides. The importance of the results for defining best practices in IPM and new approaches in studying sub-lethal effects on *M. pygmaeus* and other hemipteran predators are discussed.

**Abstract:**

In this study, the effects of paraffin oil, copper hydroxide, copper oxychloride, wettable sulfur, *Beauveria bassiana* and deltamethrin, as a toxic reference treatment, on the survival and predation rate of *M. pygmaeus* were investigated. In each treatment, the prey were classified as slightly, partially or fully consumed. The mortality rate after contact exposure was high (66.6%) when nymphs were treated with copper hydroxide but much lower after residual exposure (6.6%). *B. bassiana* caused 53.3% and 46.6% mortality via contact and residual exposure, respectively. The total prey consumption was significantly lower in the pyrethroid reference treatment control and *B. bassiana* treatments. The highest percentage of slightly consumed prey was recorded in the toxic reference and *B. bassiana* treatments, that of partially consumed prey in the copper hydroxide treatment and, finally, that of fully consumed prey in the paraffin oil treatment. Therefore, assessing the sub-lethal effects by separating the prey killed into slightly, partially and fully consumed is a sensitive approach to detect impacts which otherwise may remain unnoticed. The results provide information for the most appropriate use of *M. pygmaeus* in IPM programs and introduce more sensitive approaches in the detection of side-effects of pesticides on *M. pygmaeus* and other hemipteran predators.

## 1. Introduction

Integrated Pest Management (IPM) in tomato crops is a complex process since many insect pests may be simultaneously present on tomato plants sharing high reproduction rates, multivoltinism and pesticide-resistance issues [1,2,3]. The invasion in Europe of the destructive tomato pest *Tuta absoluta* (Meyrick) (Lepidoptera: Gelechiidae) and its expansion worldwide has caused serious difficulties in IPM in tomato crops [4,5,6]. To overcome these shortcomings, together with the controversial effects of synthetic pesticides on human and environmental health, the use of selective or low-risk pesticides has been prioritized [7]. However, although they are assumed to be more environmentally benign in comparison to broad-spectrum pesticides, their profiles may not always be compatible with the use of natural enemies [8,9,10]. 

Generally, the integration of natural enemies with pesticide use is a challenging issue and requires detailed assessment of the lethal and sub-lethal effects of pesticides. In fact, sub-lethal effects may significantly interfere with the physiology or the behavior of natural enemies, lowering their potential to suppress pest populations [11,12,13,14,15]. Adverse effects on predation rates may directly reduce the efficacy of natural enemies in biological control. Hence, among sub-lethal effects, those affecting predatory behavior and prey consumption are of fundamental importance in assessing the compatibility of a predator with the use of a pesticide within the IPM context [16].

The omnivorous predator *Macrolophus pygmaeus* (Rambur) (Hemiptera: Miridae) is a main tool in the biological control of *T. absoluta*, whiteflies and other pests of tomatoes, used in augmentative releases or in conservation biological control schemes [4,17,18]. The sub-lethal effects on its prey consumption were found to be significant when treated with thiacloprid [12] or chlorantraniliprole [19]. Therefore, although this species is commonly used, the side-effects of relatively few insecticides on its predation rate have been studied.

In addition to insecticides, fungicides are applied repeatedly in tomato crops, particularly in the period of fast plant growth which coincides with the period of *M. pygmaeus* release and establishment [20,21]. Martinou et al. [12] showed that copper hydroxide residues caused significant mortality in *M. pygmaeus* nymphs. Copper oxychloride caused 30% mortality in the closely related predator *Nesidiocoris tenuis* (Reuter) (Hemiptera: Miridae) when plants, predators and food had been sprayed; however, when only exposed through dried residues, the mortality of the predator was similar to that of the control [22]. Therefore, the side-effects depend on the exposure method; however, our knowledge of the lethal, and mainly the sub-lethal, effects of fungicides on *M. pygmaeus* is very limited. 

Regarding its predatory behavior, previous studies have revealed that, interestingly, *M. pygmaeus* may slightly, partially or totally/fully consume its prey [23,24,25]. Generally, a higher number of prey are partially consumed when more prey are available or when prey that are larger in size are used [23,24,25]. However, the association of this diverse predatory behavior with a stressor such as the application of a pesticide has not been examined. Taking into account the effects of pesticide use on each of these predatory behaviors may be more informative than assessing only the effects on total predation. This approach may be particularly relevant in cases in which strong lethal effects are not expected, such as when pesticides registered in organic farming are under evaluation. 

In the current study, we addressed the lethal effects of organic-production-approved insecticides and fungicides on *M. pygmaeus* and, for the first time, the effects of insecticides and fungicides on the prey consumption rate of *M. pygmaeus* by distinguishing the killed prey as slightly, partially or fully consumed under different pesticide application scenarios. 

## 2. Materials and Methods

### 2.1. Biological Material

The colony of *M. pygmaeus* was initiated from nymphs and adults (Miridbug™, Koppert B.V., BE Berkel en Rodenrijs, The Netherlands) and kept on eggplants (cv. Bonica F1, General Fytotechniki S.A., Athens, Greece) with food that was a mixture of *Ephestia kuehniella* Zeller (Lepidoptera: Pyralidae) eggs and cysts of *Artemia* sp. (Crustaceae) (Entofood™, Koppert B.V., The Netherlands) offered *ad libitum* at 3d intervals. The culture was kept in wooden entomological cages (80 cm length × 80 cm width × 70 cm height) under greenhouse conditions.

In the experiments, tomato plants (cv. Elpida F1, Spirou House of Agriculture, Athens, Greece) were used. The plants were grown in plastic seed trays with compost (Bas Van Burren B.V., The Netherlands) and then transplanted to pots. The plants were kept in entomological cages in an air-conditioned glasshouse at 25 ± 3 °C and 65 ± 10% RH. No pesticide was applied to the plants. 

### 2.2. Experimental Setup

The effects of fresh residues through contact with contaminated surfaces without and with direct exposure (direct contact with the toxic compound) on the survival and the predation rate and behavior of *M. pygmaeus* nymphs were examined. The first scenario represented the release of the predator soon after the spray application and the second when the predator has colonized the plants before the spray application (i.e., the worst-case scenario). 

In the assays, 5th-instar nymphs of the predator were used. These nymphs developed from 3rd-instar nymphs collected from the colony and were placed in Petri dishes (15 cm in diameter and 1.5 cm in height) with a mesh-covered hole in the lid (3 cm in diameter) for ventilation. In each dish, tomato leaflets and an abundant food supply (Entofood™) were added. The dishes were kept at 25 ± 1 °C and 65 ± 5% RH, and a photoperiod of 16:8 (L:D) h was used. When they reached the 5th instar, the nymphs were transferred individually in a dish (9 cm in diameter) with only a tomato leaflet and soaked cotton as a water source. After 24 h, the nymphs were used in the experiments. 

The pesticides used were paraffin oil, wettable sulfur, copper hydroxide, copper oxychloride and *Beauveria bassiana* (Table 1). All were organic-production-approved. The pesticides were evaluated by applying their maximum label rates for tomato greenhouse crops, according to the Phytosanitary Products Registry of the Hellenic Republic Ministry of Rural Development and Food. As a toxic reference treatment, deltamethrin (Decis 2.5 EC, Bayer S.A.S., Lyon, France) was used. This insecticide was selected due to its wide use in pest control [26]. As a negative control, distilled water was used. Distilled water was used for the preparation of the solutions as well. 

The mortality and the prey consumption of the nymphs were recorded after their spraying (contact exposure) or exposure to dry residues. After starvation (as described before), each nymph was placed singly on a tomato leaflet and sprayed with a hand trigger sprayer. The nozzle of the sprayer was set to the mist position and directed from a distance of 0.2 m towards the leaflet, thus wetting the nymph uniformly. By a similar methodology, the *E. kuehniella* eggs (EPHEScontrol™, Agrobio S.L., Almeria, Spain) used as prey were sprayed. Prior to use, each egg was carefully examined under a stereomicroscope, and eggs with irregular shapes or colors were discarded. After spraying, the sprayed nymphs and eggs were dried at room conditions for 30 min. 

Each leaf of the tomato plants ca. 35–40 cm in height was immersed in one of the respective solutions for 5 s [29]. The leaves of each plant were immersed in only a single solution. After 30 min, one tomato leaflet of the middle leaf of the plant was carefully cut. Then, the leaflet was placed in a dish (9 cm in diameter, as described before) upside down and its petiole was wrapped in a piece of cotton moistened with distilled water. After that, 50 eggs sprayed with the respective solutions were placed on it, evenly distributed. The leaflets used were of a similar size to each other. In each dish, a single nymph (either sprayed or not) was introduced. A single nymph was used with the aim of counting the predation rate per nymph and to avoid effects of intraspecific interactions. Then, the dish was sealed with paraffin film and maintained at 25 ± 1 °C and 65 ± 5% RH with a photoperiod of 16:8 (L:D) h. The plants were kept at indoor conditions.

The prey consumption of each nymph was recorded after 24 h (i.e., 1 day post-treatment, 1 dpt). The consumed eggs were distinguished into three sub-categories, as confirmed by our preliminary observations showing that sucked eggs could be distinguished into: slightly (<10%), partially (40–60%) and fully (>90%) sucked. In preliminary experiments, eggs sprayed with the substances did not change in shape after 24 h. Then, the leaflet was replaced with one that was cut off from a plant whose leaves had been immersed in the respective pesticide solution on 0 dpt, as described above. On the leaflet, unsprayed food was added, i.e., *E. kuehniella* eggs and *Artemia* cysts, *ad libitum*. The survival of the nymph was recorded at 1, 4 and 7 dpt. The leaflet and the prey were replaced again at 4 dpt. A nymph was considered dead if did not move after being prodded with a bristle. The bioassays were carried out according to a completely randomized design. Fifteen replicates were used per treatment. 

### 2.3. Statistical Analyses

The mortality of the nymphs at 7 dpt was compared between residual- and contact-exposed scenarios for each pesticide by the log-rank procedure, used to compare the respective survival curves. In the negative control treatment, where distilled water was used, the data on the percentages of the preyed eggs found slightly, partially or totally consumed were analyzed by a 1-way ANOVA with the factor “contact vs. exposed to residuals nymph” for each of the slightly, partially or totally consumed prey, after being arcsine transformed. The data on the total predation rate (i.e., the sum of the eggs found slightly, partially and totally consumed per predator) were analyzed by a 2-way ANOVA with the factors “contact vs. exposed to residuals nymph” and “pesticide treatment” after the data were log transformed. The data on the percentage of eggs found slightly, partially or totally consumed of the total number of eggs preyed on were analyzed by separate 2-way ANOVAs with the factors “contact vs. exposed to residuals nymph” and “pesticide treatment” after the data were arcsine transformed. In all cases, the means were separated by the Tukey–Kramer HSD test (a = 0.05). Statistical analyses were performed with the statistical package JMP [30].

The mortality rates were used to classify the compounds according to the recommendations of the International Organization for Biological and Integrated Control of Noxious Animals and Plants (IOBC): substances that caused mortality <30% were classified as harmless, those that caused 30 to 79% mortality as slightly harmful, those that caused 80 to 99% mortality as moderately harmful and, finally, those that inflicted mortality >99% as harmful [31]. 

## 3. Results

### 3.1. Lethal Effects

**Figure 1:**
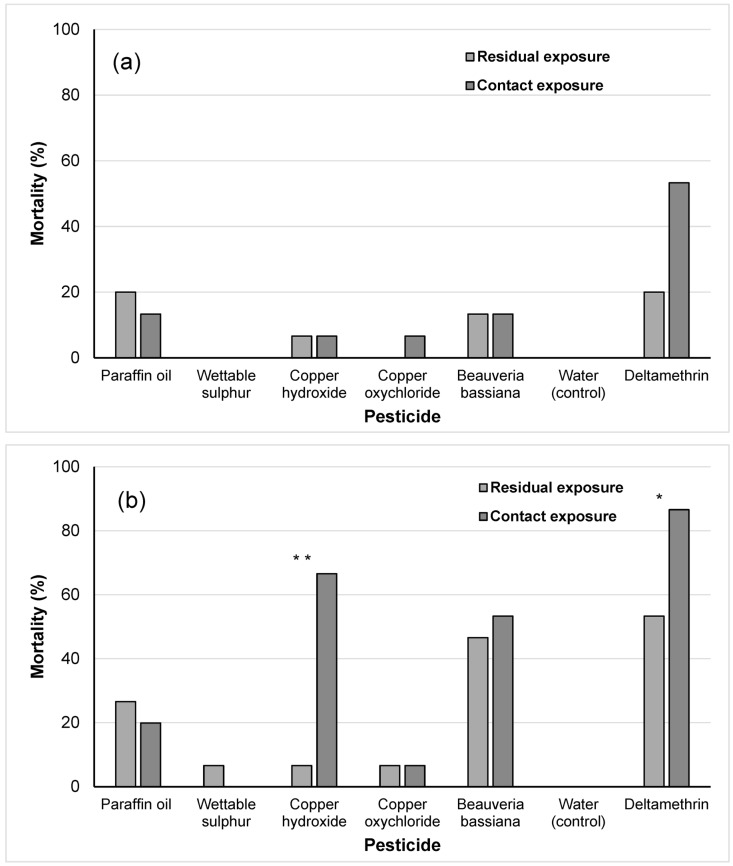
Percentage mortality caused by insecticides and fungicides in *Macrolophus pygmaeus* nymphs by contact and residual exposure after 1 (**a**) and 7 (**b**) days. Asterisks indicate significant differences between contact- and residual-exposed nymphs within each pesticide treatment (** *p* < 0.01, * *p* < 0.05, log-rank procedure).

Nymphal mortality at 7 dpt was highest in the case of the toxic reference treatment, which reached 53.3% for the nymphs exposed to dry residues and 86.6% for the nymphs that were sprayed. In the negative control treatment, no mortality was recorded (Figure 1a,b). Among the other pesticides, in the nymphs exposed to residuals, mortality at 7 dpt was highest (46.6%) after the intervention with *B. bassiana*, followed by that recorded for paraffin oil (26.6%). The mortality rate for the contact-exposed nymphs was highest after the application of copper hydroxide (66.6%), followed by *B. bassiana* (53.3%). Paraffin oil caused 19.9% mortality, whereas sulfur and copper oxychloride caused negligible mortality. A large difference was observed between the mortalities of contact- and residual-exposed nymphs in the case of copper hydroxide (66.6% vs. 6.6%). The comparison of the survival curves for the two exposure bioassays for copper hydroxide at 7 dpt showed a significantly stronger effect in the case of the contact-exposed nymphs than in those exposed to residues (*χ^2^*_1_ = 78.9, *p* < 0.004). A similar significant effect was also recorded for the case of deltamethrin (*χ^2^*_1_ = 4.9, *p* < 0.032). The mortality caused by the copper hydroxide was due to the incomplete removal of the exuviae from the hind legs of the emerging adult during moulting. 

According to the IOBC classification, deltamethrin was classified as moderately harmful to sprayed nymphs and slightly harmful to nymphs exposed to residuals. *B. bassiana* was classified as slightly harmful for both application scenarios. Copper hydroxide was classified as slightly harmful when the nymphs had been sprayed. Paraffin oil, wettable sulfur and copper oxychloride were classified as harmless.

### 3.2. Sub-Lethal Effects

In the negative control treatment, where nymphs were sprayed or not with distilled water and the leaflet and prey were sprayed with distilled water too, the effect of contact vs. residual exposure on nymphs was not statistically significant for the slightly, partially or totally consumed prey (F_1,26_ = 3.08, *p* > 0.09; F_1,26_ = 0.36, *p* > 0.55; F_1,26_ = 1.26, *p* > 0.27, respectively). The predator showed a significantly higher tendency to consume the eggs totally (73.83 ± 4.30%) than slightly (18.97 ± 2.80%) or partially (7.43 ± 2.04%).

**Figure 2:**
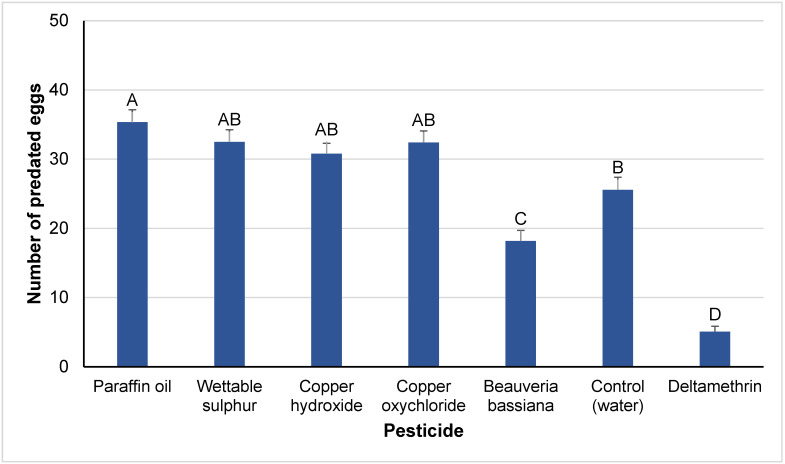
Total egg predation (means ± SEs) of *M. pygmaeus* nymphs exposed to contact or residual effects of insecticides and fungicides, 24 h after their treatment. Columns followed by the same capital letter are not significantly different among pesticide treatments (ANOVA, HSD test, *p* < 0.05).

The total predation rate was affected by the pesticide treatment (F*_6,194_* = 71.28, *p* < 0.001), whereas the exposure route and the exposure route x pesticide interaction were not significant (F*_1,194_* = 0.02, *p* > 0.95; F*_6,194_* = 1.64, *p* > 0.13, respectively) (Figure 2). Among all the treatments, the total prey consumption was highest in the treatment with paraffin oil, being significantly higher than that in the negative control. In the other treatments, significant differences were not recorded in comparison to each other and the negative control, except for *B. bassiana*, for which the total prey consumption was significantly lower than in the negative control, and deltamethrin, for which it was significantly lower than in all the other treatments, including those of *B. bassiana* and the negative control. 

**Figure 3:**
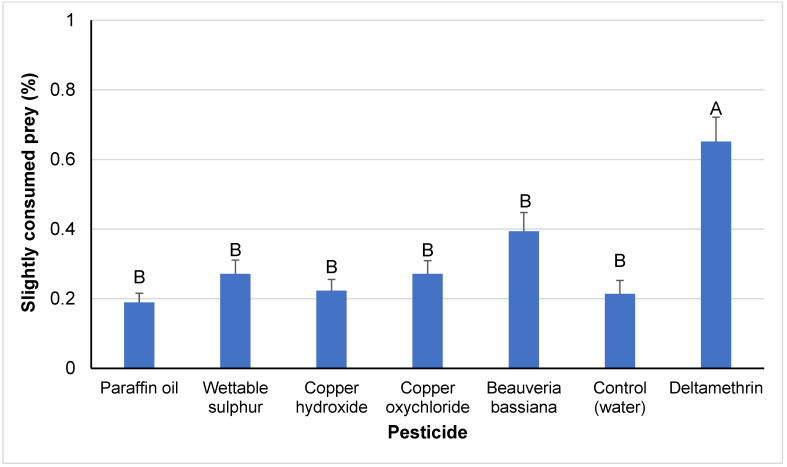
Percentages of slightly consumed prey (means ± SEs) by *M. pygmaeus* nymphs exposed to contact or residual effects of insecticides and fungicides, 24 h after their treatment. Columns followed by the same capital letter are not significantly different (ANOVA, HSD test, *p* < 0.05).

The percentage of slightly consumed eggs was significantly affected by pesticide treatment (F*_6,193_* = 11.88, *p* < 0.001), whereas the exposure route and pesticide interactions were not significant (F_1,193_ = 2.57, *p* > 0.11; F*_6,193_* = 1.99, *p* > 0.07, respectively). Among the pesticides used, the highest percentage of slightly consumed prey (65%) was recorded in the case of the toxic reference treatment (Figure 3). This was followed by a significantly lower value recorded for the *B. bassiana* treatment, which, however, was not significantly different from all the other treatments, among which significant differences were not recorded. 

**Figure 4:**
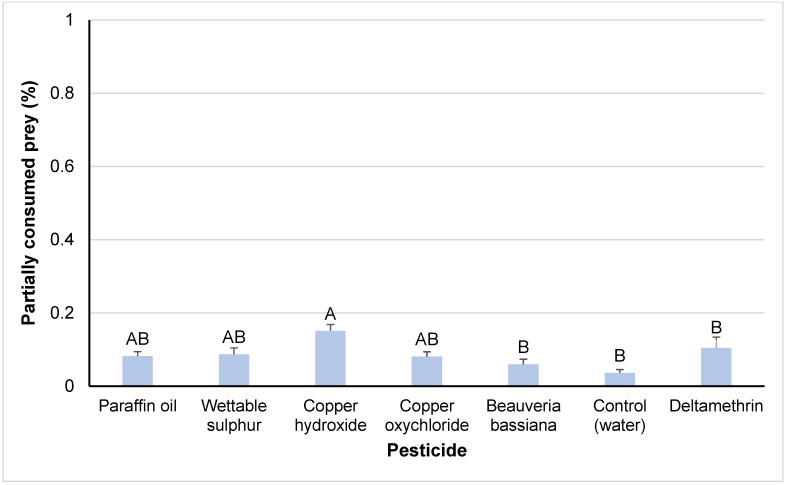
Percentages of partially consumed prey (means ± SEs) by *M. pygmaeus* nymphs exposed to contact or residual effects of insecticides and fungicides, 24 h after their treatment. Columns followed by the same capital letter are not significantly different (ANOVA, HSD test, *p* < 0.05).

The effects of “pesticide treatment” and “contact vs. residual exposed nymph” on the percentage of partially consumed eggs were significant (F*_6,193_* = 6.12, *p* < 0.001; F*_1,193_* = 6.67, *p* < 0.01, respectively), whereas their interaction was not significant. Among the pesticides, the highest percentage was recorded in the case of copper hydroxide, which differed significantly from the negative control, *B. bassiana* and deltamethrin (Figure 4). Among paraffin oil, copper oxychloride and wettable sulfur, significant differences were not recorded. Contact-exposed nymphs had a significantly higher tendency to consume the prey partially than the nymphs exposed to residuals. 

**Figure 5:**
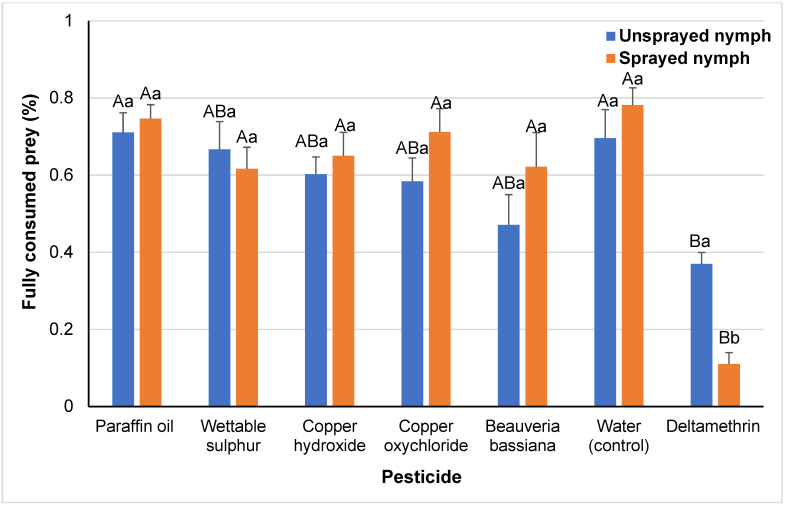
Percentages of fully consumed prey (means ± SEs) by *M. pygmaeus* nymphs exposed to contact or residual effects of insecticides and fungicides, 24 h after their treatment. Columns followed by the same capital letter are not significantly different among treatments for the residual- or contact-exposed nymphs, whereas columns followed by the same small letter are not different between residual- and contact-exposed nymphs, within each pesticide treatment (ANOVA, HSD test, *p* < 0.05).

The percentage of totally consumed eggs was affected by the “pesticide treatment” (F*_6,193_* = 13.40, *p* < 0.001) and the effect of the factor “contact vs. residual exposed nymph” was not significant (F*_1,193_* = 0.15, *p* > 0.69), but their interaction had a significant effect (F*_6,193_* = 2.31, *p* < 0.035). Among the pesticide treatments, the highest percentages were recorded for the negative control and paraffin oil. However, significant differences were not recorded among the pesticide treatments, except for the case of the toxic reference treatment, where the percentage of totally consumed prey was significantly lower than in the negative control and paraffin oil treatments for the residual-exposed nymphs, whereas in the case of the contact-exposed nymphs this percentage was significantly lower than for all the other pesticides (Figure 5). In the case of the toxic reference treatment, the sprayed nymphs showed a significantly lower tendency to fully consume the prey than the unsprayed nymphs.

## 4. Discussion

### 4.1. Lethal Effects

Among the insecticides tested, *B. bassiana* was ranked as slightly harmful to *M. pygmaeus*. In a previous study, the survival rate of second-instar nymphs was not significantly affected in comparison to the control treatment 2 weeks after direct exposure or exposure to contaminated prey [32]. Probably, there is variation in the sensitivity of nymphal instars to *B. bassiana*. In support of this, it has been reported that the sensitivity of older nymphs of the whitefly *Trialeurodes vaporariorum* (Westwood) was higher than that of young nymphs [33].

The paraffin oil treatment was classified as harmless. Although it is a commonly used insecticide in vegetable crops, its lethal or sub-lethal effects have not been studied in *M. pygmaeus*. Angeli et al. [34] tested the effect of a mineral oil formulation on the survival of fourth-instar nymphs of the predator *Orius laevigatus* (Fieber) (Hemiptera: Anthocoridae) exposed to residues. The oil caused 8.3% mortality and was classified as harmless. Biondi et al. [9] reported that paraffin oil was slightly harmful (40%) to *O. laevigatus* adults when exposed at the highest recommended dose under laboratory conditions to 1 h old pesticide residues; however, in the case of 14 d old residues, the mortality was reduced (20%). 

Copper hydroxide proved harmless to nymphs under residual exposure. In contrast, the sprayed nymphs showed an increased mortality as high as 66.6%, classifying this fungicide as slightly harmful. According to our observations, this mortality was due to the incomplete removal of the exuviae of the newly emerged adults. A similar phenomenon has been reported for *Empoasca fabae* (Harris) (Hemiptera: Cicadellidae) after treatment with Bordeaux mixture [35]. Martinou et al. [12] showed that copper hydroxide caused 58% mortality at 72 h in fifth-instar nymphs of *M. pygmaeus* which were sprayed and fed on sprayed eggs on a sprayed tomato leaflet. However, testing contact vs. residual exposure enabled us to clarify that the nymphs were affected only by contact exposure; otherwise, the effect of copper hydroxide was negligible. Copper oxychloride was classified as harmless, causing a mortality rate of 6.6%. A relevant study has not been conducted for *M. pygmaeus*. In contrast, this substance caused as high as 30% mortality in *N. tenuis* [22] but has been reported as harmless for the nymphs of *O. laevigatus* [34]. 

Spraying with wettable sulfur caused a negligible mortality in *M. pygmaeus*. *N. tenuis* mortality was increased when adults were placed on plants immediately after the application of wettable sulfur; however, this effect was not different to the control [36]. This substance caused 50% mortality in nymphs of *Deraeocoris brevis* (Uhler) (Hemiptera: Miridae) and after longer developmental times [29]. According to Van de Veire et al. [37], wettable sulfur caused mortality of 25% under laboratory and 30% under greenhouse conditions in sprayed adults of *O. laevigatus* that foraged on sprayed plants and fed on sprayed food. In another study, wettable sulfur caused 28% mortality in nymphs of *O. laevigatus* exposed to fresh residues [34].

Finally, deltamethrin was classified as moderately harmful to nymphs that were sprayed and slightly harmful to nymphs exposed to residuals. In another study, the effect of deltamethrin on *N. tenuis* was found to be much lower when exposed only to contaminated prey (2.7%) [38]

### 4.2. Sub-Lethal Effects

Our results for the negative control treatment showed that almost 27% of the prey items killed by *M. pygmaeus* were slightly or partially consumed. In previous studies in which aphids had been used as prey, the percentage of partial prey consumption of this predator reached 23% [23,24], whereas when *Acyrthosiphon pisum* (Harris) (Hemiptera: Aphididae) was used as prey, it was even higher [25]. This high percentage of slightly or partially consumed prey indicates that separating the prey killed into totally, partially or slightly consumed constitutes a useful tool in monitoring the effects on the predatory behavior of this predator caused by pesticides. 

In the treatments with deltamethrin, the nymphs of *M. pygmaeus* showed the lowest predation rate among all the pesticides tested. In addition, nymphs treated with deltamethrin showed the highest percentage of slightly consumed eggs and, on the other hand, the lowest percentage of totally consumed eggs. These results indicate a lesser ability of the nymphs to locate their prey and then to complete consumption of a prey item, as they are liable abandon it soon after the initiation of feeding. It is likely that this is due to the lack of locomotor coordination that has been reported as one of the first symptoms in insects treated with pyrethroids [39,40]. In concordance with these results, Wanumen et al. [38] reported a negligible effect of contaminated prey on the predation rate of *N. tenuis*, showing that the effect recorded in our work is most likely due to contact of the predator with the insecticide. *Orius insidiosus* (Say) (Hemiptera: Anthocoridae) adults contaminated topically with an LC_20_ solution of deltamethrin showed a high reduction in their predation rate [13]. 

Following the application of *B. bassiana*, the total predation rate was significantly lower than in the negative control, whereas the frequency of slightly consumed prey was relatively increased in comparison to the negative control. Labbè [41] showed that the predator *Dicyphus hesperus* Knight (Hemiptera: Miridae) avoided consuming prey sprayed with this fungus, likely because the infection of the prey may lead to the production of toxins or other changes impacting prey quality. *Phytoseiulus persimilis* Athias-Henriot (Acari: Phytoseiidae) displayed a significant avoidance response to *B. bassiana* on bean leaves [42]. In another study, the consumption by *D. hesperus* of sprayed *E. kuehniella* eggs treated with the entomopathogen *Paecilomyces fumosoroseus* (Ascomycota: Hypocreales) was significantly reduced [43]. Meyling and Pell [44] showed that adults of *Anthocoris nemorum* (L.) (Hemiptera: Anthocoridae) did not prefer and spent significantly reduced foraging time on leaves sprayed with *B. bassiana*. The results of our work agree with the results of the previously mentioned studies showing adverse effects of this fungus on the prey consumption rates of several predators. The results of our study showed, further, that the predator’s feeding process was frequently interrupted, leaving the prey little consumed, likely due to deterioration in prey quality after they were sprayed with the fungus. 

Interestingly, *M. pygmaeus* nymphs sprayed with paraffin oil showed the highest prey consumption. This effect may be due to changes that are caused by the spraying on the behavior of the nymph or the quality of the prey, a phenomenon that needs further evaluation. 

Among the pesticides tested, the highest percentage of partially consumed prey was recorded in the case of copper hydroxide. Therefore, this fungicide interfered with the predatory behavior of *M. pygmaeus* and, thus, in addition to the high mortality it causes in sprayed nymphs, it may also reduce the efficacy of the predator in pest control due to sub-lethal effects.

## 5. Conclusions

This work showed that *B. bassiana* may cause increased mortality in *M. pygmaeus* nymphs, as may copper hydroxide when used after the release of *M. pygmaeus*; *B. bassiana* increased the proportion of slightly consumed prey, copper hydroxide increased that of partially consumed prey and paraffin oil caused increased total prey consumption, depending on the application scenario. The selectivity of pesticides may be due to many factors (i.e., [45]), and thus studying effects on slightly, partially or fully consumed prey may increase the sensitivity in our approaches to detect the sub-lethal effects of pesticides in studying side-effects on predators which show partial prey consumption, such as the hemipteran ones (i.e., [25,46,47,48]).

## Figures and Tables

**Table 1:** Details of the organic-production-approved pesticides [27,28] and the concentrations used in the bioassays of the current study.

Common Name/a.i.	Mode of Action	Chemical Class	Brand Name/Formulation	Manufacturer	Highest Label Rate g a.i./L
Paraffin oil-Mineral oil	NC	Diverse	Nitropol O EW	W. Neudorff, Athens, Emmerthal, Germany	5.46
Sulfur	M2	Inorganic	Frame 80 WG	Agrostulln, Stulln, Germany	2.4
Copper hydroxide	M1	Inorganic	Champ 36 SC	Nufarm, Melbourne, Austria	1.27
Copper oxychloride	M1	Inorganic	Cuprachlor 50 WP	Industrias Quimicas del Vallès, S.A, Barcelona, Spain	1.25
*Beauveria bassiana*	UNF	Entomopathogen	Metab SL	Microspore-Sacom, Greece	11.5 × 10^7^ conidia
Deltamethrin	3A	Pyrethroid	Decis 2.5 EC	Bayer S.A.S, Lyon, France	0.75

## Data Availability

Data supporting results can be found at: http://hdl.handle.net/10329/8076.
